# Reliability of standard pupillometry practice in neurocritical care: an observational, double-blinded study

**DOI:** 10.1186/s13054-016-1239-z

**Published:** 2016-03-13

**Authors:** David Couret, Delphine Boumaza, Coline Grisotto, Thibaut Triglia, Lionel Pellegrini, Philippe Ocquidant, Nicolas J. Bruder, Lionel J. Velly

**Affiliations:** Neurocritical Care Unit, University Hospital Saint Pierre, Réunion University, BP 350, Saint Pierre, 97448 la Réunion France; Inserm, UMR 1188 Diabète athérothrombose Thérapies Réunion Océan Indien (DéTROI), plateforme CYROI, 2 rue Maxime Rivière, Sainte Clotilde, 97490 la Réunion France; Department of Anesthesiology and Critical Care Medicine, University Hospital Timone, Aix Marseille University, 264 rue St Pierre, Marseille, 13005 Bouches du rhone France

**Keywords:** Pupillometer, Pupillary light reflex, Pupillary reactivity, Anisocoria, Pupillary size, Neurocritical Care, Neurological examination

## Abstract

**Background:**

In critical care units, pupil examination is an important clinical parameter for patient monitoring. Current practice is to use a penlight to observe the pupillary light reflex. The result seems to be a subjective measurement, with low precision and reproducibility. Several quantitative pupillometer devices are now available, although their use is primarily restricted to the research setting. To assess whether adoption of these technologies would benefit the clinic, we compared automated quantitative pupillometry with the standard clinical pupillary examination currently used for brain-injured patients.

**Methods:**

In order to determine inter-observer agreement of the device, we performed repetitive measurements in 200 healthy volunteers ranging in age from 21 to 58 years, providing a total of 400 paired (alternative right eye, left eye) measurements under a wide variety of ambient light condition with NeuroLight Algiscan pupillometer. During another period, we conducted a prospective, observational, double-blinded study in two neurocritical care units. Patients admitted to these units after an acute brain injury were included. Initially, nursing staff measured pupil size, anisocoria and pupillary light reflex. A blinded physician subsequently performed measurement using an automated pupillometer.

**Results:**

In 200 healthy volunteers, intra-class correlation coefficient for maximum resting pupil size was 0.95 (IC: 0.93-0.97) and for minimum pupil size after light stimulation 0.87 (0.83–0.89). We found only 3-pupil asymmetry (≥1 mm) in these volunteers (1.5 % of the population) with a clear pupil asymmetry during clinical inspection. The mean pupil light reactivity was 40 ± 7 %. In 59 patients, 406 pupillary measurements were prospectively performed. Concordance between measurements for pupil size collected using the pupillometer, versus subjective assessment, was poor (Spearmen's rho = 0.75, IC: 0.70-0.79; *P* < 0.001). Nursing staff failed to diagnose half of the cases (15/30) of anisocoria detected using the pupillometer device. A global rate of discordance of 18 % (72/406) was found between the two techniques when assessing the pupillary light reflex. For measurements with small pupils (diameters <2 mm) the error rate was 39 % (24/61).

**Conclusion:**

Standard practice in pupillary monitoring yields inaccurate data. Automated quantitative pupillometry is a more reliable method with which to collect pupillary measurements at the bedside.

**Electronic supplementary material:**

The online version of this article (doi:10.1186/s13054-016-1239-z) contains supplementary material, which is available to authorized users.

## Background

Evaluation of pupil size and its light reflex mechanism is an integral part of the protocol for the treatment and management of severely brain-injured patients in ICUs worldwide [1]. The clinical implications for detection of any abnormality have been firmly established in terms of neurological outcome [2–4], morbidity, and mortality [5]. Measurements of pupil size and reactivity are considered to be of great prognostic importance for patients with critical conditions. Given their indispensible role in establishing the severity of injury and/or illness, it is not surprising that there is considerable interest in how these parameters may be measured accurately [6]. In a detailed study correlating changes in pupil shape and intracranial pressure (ICP), Marshall et al. [7] described changes in pupil shape in a number of patients with an ICP lower than 20 mmHg. The association of these abnormalities with posterior frontal and temporal lesions would suggest that other changes in pupillary function might also be detected, even when the ICP is normal, particularly for lesions adjacent to the brainstem.

Pupillary evaluation in the clinical setting is often performed in a subjective manner, with a penlight for reactivity and a pupil gauge for pupil size. Common terminologies employed in the clinical literature to describe the pupillary light reflex (PLR) and pupil size include the following: unilateral, bilateral-nonreactive, fixed, dilated, brisk, sluggish, and nonreactive. These subjective terms are often applied in the absence of any standardized clinical protocol or definition. The potential therefore exists to compound inaccuracies and inconsistencies, especially given substantial inter-examiner variability [8]. Moreover, ambient light conditions can impact the validity of visual assessments of the pupil, and exacerbate inter-observer disagreement. As shown recently, these discrepancies can be as high as 40 % [9]. Infrared pupillometry is a notable exception because this provides an objective measurement of the PLR [10].

While several quantitative pupillometer devices have been developed, these are still used primarily in research settings, either because the first devices were relatively cumbersome or because they were simply ill-suited to the clinical care environment [11]. The recent development of portable, hand-held infrared pupillometers represents the first innovative step in introducing this more reliable technology to the clinic [12]. The objective of this prospective, observational, double-blinded study was to compare the efficacy of standard versus automated pupillary measurements for pupil size evaluation, anisocoria detection, and PLR in brain-injured patients. These data would allow us to gauge whether standard pupillometry, as currently practiced, is of sufficient accuracy for our clinical practice.

## Methods

### Study design

After obtaining ethical committee approval (Comités de Protection des Personnes, Marseille, France; number 2012-1270), this prospective, observational, double-blinded study was conducted in two neurocritical care units (NCCUs; Marseille and Saint-Pierre la Reunion, France). We used a portable pupillometer (NeuroLight Algiscan; IDMED, Marseille, France) to measure pupil diameter and reactivity. Both sets of experimenters, the nurses performing the standard pupillary examination, and the physicians performing the electronic pupillometer measurements were all blinded to each other’s data.

### Study population

We included patients over 18 years old admitted to the NCCU within 48 hours of an acute brain injury (head trauma, subarachnoid hemorrhage, stroke, neurosurgical complications after surgery, medical intracranial hypertension). We excluded all patients with a direct eye trauma, as well as any with a medical history that could impact the relevance of eye examination (opalescent cataract, iris surgery, blindness, third intracranial nerve direct damage). Patients with irreversible signs of coma at admission (bilateral and nonreactive mydriasis) were also excluded. In order to determine inter-observer agreement and repeatability of the device, 200 healthy volunteers who had no history of intracranial or ophthalmological disease were also studied under a wide variety of ambient light conditions by four physicians (two neurointensivist seniors (LJV, DC) and two residents (DB, CG)).

### Standard, visual, pupil parameters

Patients were examined by 10 nurses with an average of 10 years of experience in assessing the pupils of neurologically impaired patients. The nurse in charge of the patient estimated the pupillary size, tested for anisocoria, and assessed PLR manually using a penlight. Pupil size, anisocoria, and reactivity were evaluated under dim light conditions and with the opposite eye covered by the examiner, according to our standard practice and protocols. Quantitative assessment included pupil size, assessed using a pupil gauge, graduated in millimeters. Anisocoria was coded qualitatively as present when a size difference of ≥1 mm [2] was recorded for the patient’s pupils. Standard PLR was coded qualitatively as absent or present; lack of reactivity was scored as absent, and reactivity was scored as present.

### Quantitative pupil parameters

The NeuroLight Algiscan is a CE-approved standalone hand-held battery-operated monocular pupillometer allowing the quantitative assessment of PLR (see Additional file 1: Figure S1A). The device contains a color liquid crystal display screen, an integral illumination source, and an infrared camera capable of recording variation at the pupillary surface at 67 frames per second. The NeuroLight Algiscan does not require any calibration by the user. Its telecentric lens eliminates the parallax error characteristic of standard lenses by having a constant, nonangular field of view, at any distance from the lens. A detachable rubber cup ensures accurate position and distance from the pupil. This adapter also encompasses the patient's orbit, placing the eye in the dark, preventing stray light from entering the eye and variation of light intensity between the measurements of the two pupils. These design features have the objective to minimize inter-observer variability in pupillary evaluation.

The pupillometer illuminates the eye of a patient with a calibrated light stimulus (320 Lux, 1 second). Its lighting system meets the terms of the international IEC 62471 safety standards.

Baseline pupil size (mm) and quantitative PLR (percentage of reduction in pupil size after light stimulation) were measured after a stabilization period (4 seconds in the dark). The pupillometer measures, after pulling a manual trigger, both pupil size and reactivity. It first measures baseline pupil size during an initial 200-millisecond latency period and then emits a pulse of light lasting 1 second, and records the pupillary response to the stimulus over the subsequent 3 seconds. Each frame of the 4.2-second high-temporal resolution video (50 frames/second) is automatically processed to evaluate the diameter of the pupil as a function of time (see Additional file 1: Figure S1C). Physicians and residents were trained to use the automated pupillometer by a physician proficient in its use. To reduce the effect of consensual reflex induced by a source of light coming from the other side of the patient, the nonmeasured eye was closed. Four measurements were performed for each patient, every 24 hours. A short (less than 5 minutes) stabilization period was allowed between the measurements taken by the nurse and the physician respectively. The data recorded were: pupil size, anisocoria (with a difference between left and right pupils of ≥1 mm), and percent PLR (with a cutoff of >15 % to define normal pupil reactivity, such as 0.3 mm in a 2 mm pupil size [13]).

### Data collection

Baseline demographic data were collected: age, sex, Glasgow Coma Scale at admission, Simplified Acute Physiology Score 2 (SAPS2) at admission, diagnoses, comorbidities, vasopressor drugs, mechanical ventilation, and opioid infusion.

### Statistical analyses

Categorical variables were expressed as absolute values and percentages, and compared by a chi-square test or Fisher’s exact test. Continuous variables, expressed as mean ± standard deviation or median (interquartile range (IQR)), were compared using a Student *t* test if they were normally distributed, and a Mann–Whitney *U* test if not. To measure agreement between raters in the healthy volunteers study, we estimated intraclass correlations for two-way mixed-effects models (given with 95 % confidence intervals (CIs)). The median coefficient of variation (CoV) and IQR were used to quantify the inter-operator variation relative to its mean. The CoV was calculated as the ratio of the standard deviation to the mean. The Wilcoxon signed-rank test was used to compare the pupil size CoV between senior and resident physicians. Association between continuous and categorical variables was tested using the Spearman’s rank correlation test. This statistical analysis was applied to the complete data set, and then independently to data sets collected for pupil sizes of <2 mm, 2–4 mm, and then >4 mm. Receiver operating characteristic (ROC) analyses were performed for the three pupil size groups. *P* <0.05 was considered significant. All statistics were carried out using SPSS version 22 (IBM, Chicago, IL, USA) except for the ROC analyses, which were performed using MedCalc 9.2 (MedCalc Software, Ostend, Belgium).

## Results

### Validation study in healthy volunteers

**Fig. 1:**
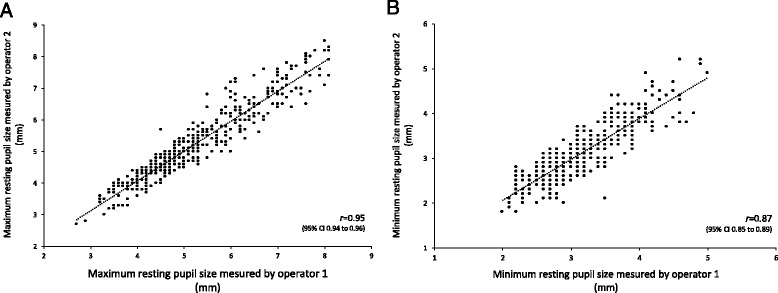
NeuroLight Algiscan’s inter-observer variability. Comparison of the maximum resting pupil size **a** and the minimum pupil size after light stimulation **b** measured with the NeuroLight Algiscan quantitative pupillometer by two operators in 200 healthy volunteers. *CI* confidence interval

Figure 1 illustrates 400 paired data measurements in 200 healthy volunteers. The mean maximum resting pupil size was 5.2 ± 1.2 mm (Fig. 1a) and the mean minimum pupil size after light stimulation was 3.2 ± 0.6 mm (Fig. 1b). The mean percentage of reduction in pupil size after light stimulation was 40 ± 7 % and in none of 800 measurements was the percentage of reduction below 15 %. The median pupillary asymmetry was –0.1 mm (IQR –0.3 to –0.2 mm) and we found a difference between right and left pupil size ≥1 mm (threshold for defining anisocoria) in only 3 of 200 healthy volunteers’ (1.5 %) measurements (see Additional file 2: Figure S2).

The mean difference between senior and junior practitioners was –0.06 ± 0.35 mm with a median CoV of 23.3 % (IQR 23.26–23.32 %). The intraclass correlation coefficient for maximum resting pupil size and minimum pupil size after light stimulation was 0.95 (95 % CI: 0.93–0.97) and 0.87 (95 % CI: 0.83–0.89), respectively.

### Patient characteristics

From January 2012 to December 2012, 59 patients were included in our study, and 406 pupillary measurements were prospectively performed. Table 1 summarizes the characteristics of patients, most of whom were assisted by mechanical ventilation (76 %), with over half (55 %) under opioid infusion (mean total daily sufentanil dose: 400 ± 50 μg).

**Table 1:** Characteristics of the patients

Demographics data	
Age, years	58 ± 18
Male, *n* (%)	35 (59)
Glasgow Coma Scale score^a^	
Median	9
Interquartile range	4-13
SAPS2	
Median	30
Interquartile range	6-56
Mechanical ventilation-assisted patients, *n* (%)	45 (76)
Opioid infusion (sufentanil), *n* (%)	32 (55)
Vasopressor infusion, *n* (%)	12 (20)
Causes of NCCU admission, *n* (%)	
Brain hemorrhage	14 (24)
Subarachnoid hemorrhage	11 (18)
Brain infarction	8 (13)
Head trauma	13 (22)
Neurosurgery	9 (15)
Meningitis	2 (4)
Cardiac arrest	1 (2)
Seizure	1 (2)

^a^Scores on the Glasgow Coma Scale range from 3 to 15, with lower scores indicating reduced levels of consciousness

*NCCU* neurocritical care unit, *SAPS2* Simplified Acute Physiology Score 2 [27]

### Comparison of pupil size evaluation methods

**Fig. 2:**
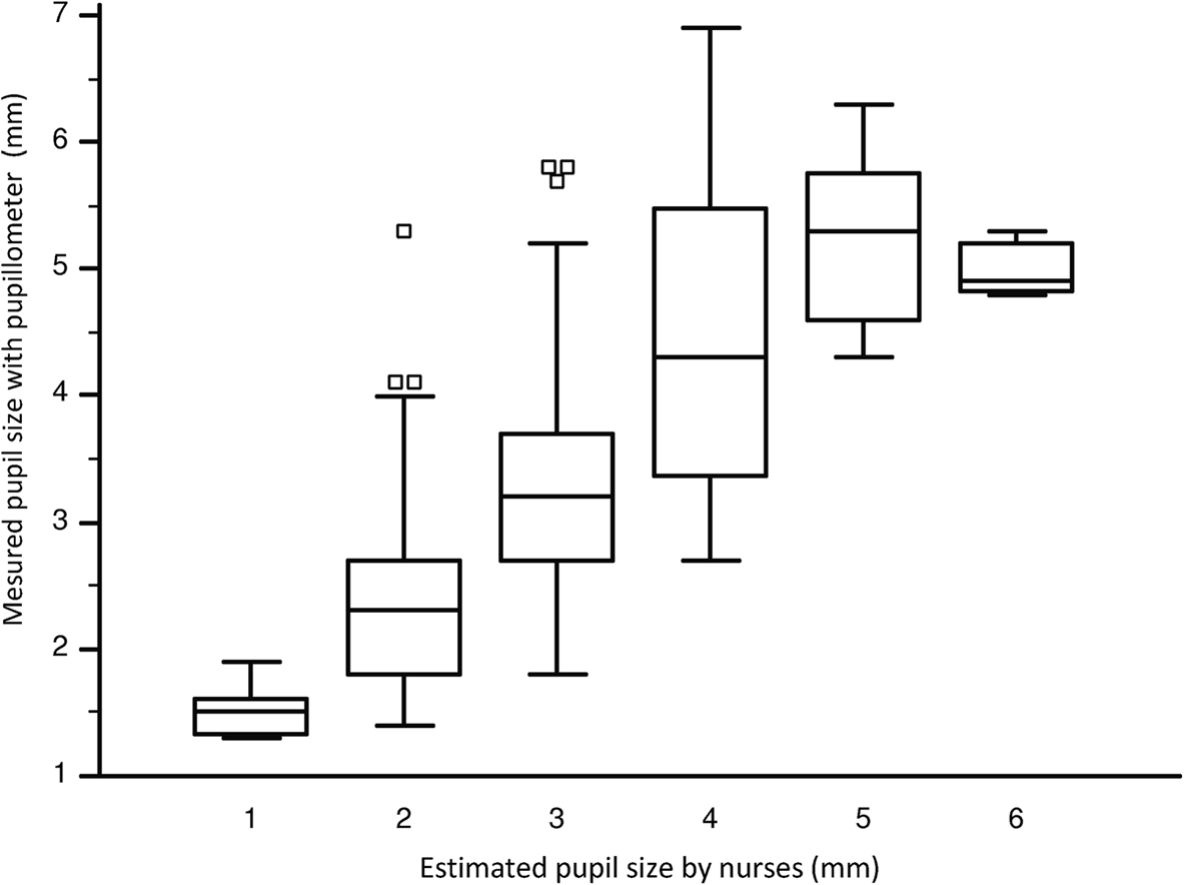
Comparison of pupil size obtained with the pupillometer versus subjective estimates. Box plots indicate medians (*horizontal line in box*), the 25th and 75th percentiles (*lower* and *upper box margins*), the 10th and 90th percentiles (*lower* and *upper error bars*), and individual patients in the lower 10th percentiles (*open squares*) for each visual measurement of pupil size. The short box whisker plots for the 1 mm and 6 mm groups suggest a high level of agreement. Box plots for the 2–5 mm groups are stretched by outliers, suggesting a lower level of agreement between the two measurements for each of these groups

**Fig. 3:**
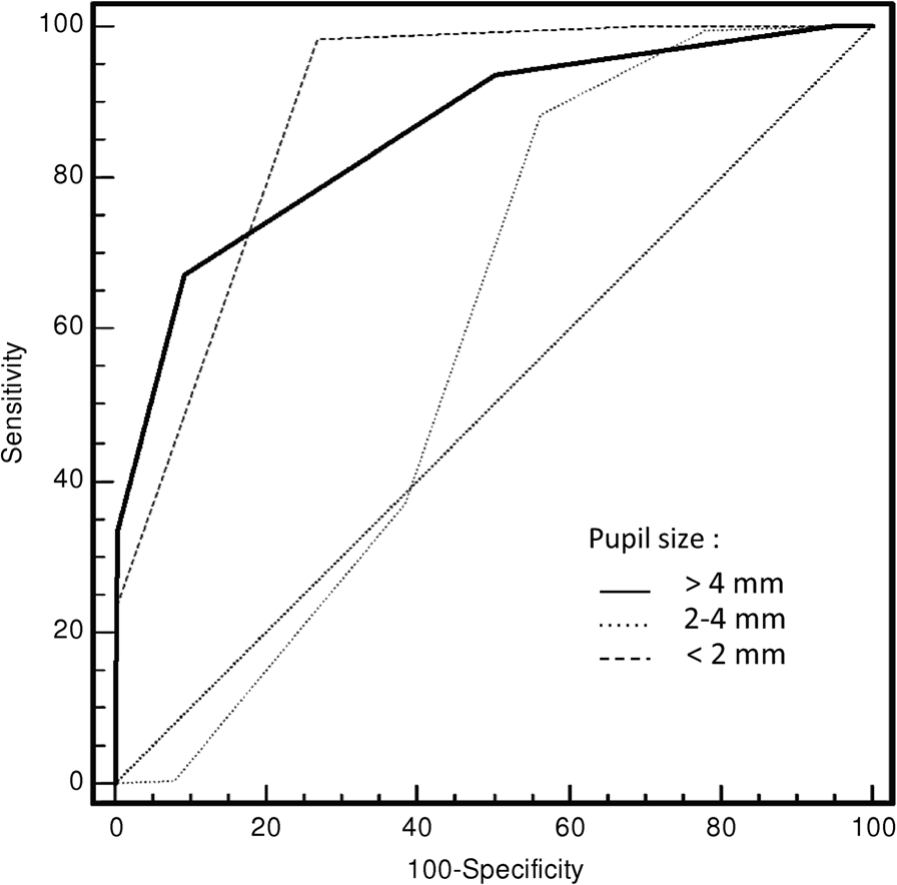
ROC curve analyses for different groups of pupil size as determined visually, and then compared with the electronic pupillometer. These data show the reliability of pupil size measurements for each pupil size group. The closer the curve approaches the 45° diagonal, the less accurate the test

The median aperture of the pupil estimated independently by standard and electronic pupillometry was 3 mm (IQR 2–4 mm) and 3.0 mm (IQR 2.3–4.3 mm), respectively. In patients receiving narcotics, we found a correlation between sufentanil dose and pupil size (Spearman’s rho = –0.36, 95 % CI: –0.48 to –0.24; *P* <0.001) (see Additional file 3: Figure S3A), but not between pupil size and midazolam or propofol doses (data not shown). Pupillometer measurements are shown alongside the nurses’ estimations of pupil size in Table 2 (Spearman’s rho = 0.75, 95 % CI: 0.70–0.79; *P* <0.001). The correlation is significant for each class of pupil size but the correlation is loose especially for small pupil size (Spearman’s rho = 0.39, 95 % CI: 0.15–0.59; *P* = 0.002). Our box plot analyses (Fig. 2) show the actual pupil size (measured using the automated pupillometer) for every measurement provided by the nursing staff. The bias between the two techniques was small (–0.36 mm), but the limits of agreement were large (–2.11 to +1.39 mm) according to the Bland–Altman method (see Additional file 4: Figure S4). The global area under the ROC curve for diagnosis of the pupil size by nursing staff was equal to 0.75 (95 % CI: 0.70–0.79). The global area under the ROC curve for diagnosis of the pupil size by the nurses in the three pupil size ranges (<2 mm; 2–4 mm; >4 mm) was respectively 0.89 (95 % CI: 0.85–0.92), 0.59 (95 % CI: 0.54–0.64), and 0.86 (95 % CI: 0.82–0.89) (Fig. 3).

**Table 2:** Spearman’s coefficient analysis between nurse and pupillometer for pupil size evaluation

Pupil size (mm)	*n*	Nurses’ estimations^a^ (mm)	Pupillometer measurements^a^ (mm)	*P* value	Spearman's coefficient (95 % CI)	*P* value
<2	61	2 (2–2)	1.8 (1.6–1.8)	0.057	0.39 (0.15–0.59)	0.002
2–4	232	3 (2–3)	2.8 (2.5–3.3)	0.005	0.44 (0.33–0.54)	<0.001
>4	113	4 (3–5)	4.9 (4.4–5.6)	<0.001	0.37 (0.19–0.51)	0.001
All	406	3 (2–4)	3.0 (2.3–4.3)	<0.001	0.75 (0.71–0.79)	<0.001

^a^Data presented as median (interquartile range)

*CI* confidence interval

### Quantitative versus standard anisocoria detection

**Fig. 4:**
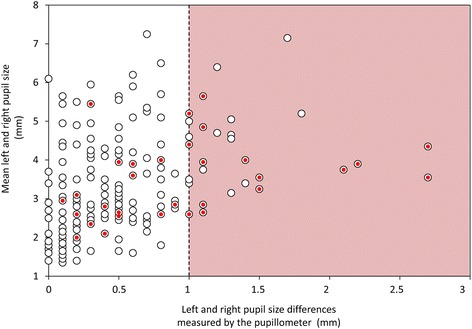
Detection of anisocoria by nursing staff. *White circles* represent for each set of paired measurements the mean pupil size according to the left and right pupil size differences measured by the pupillometer. Anisocoria was defined as a pupil size difference of ≥1 mm (*red square*). *Red circles* represent anisocoria detected by the nurses. Nursing staff failed to diagnose half of the cases (15/30) of anisocoria detected using the pupillometer device and wrongly detected 16 episodes of anisocoria (Color figure online)

Using our ≥1 mm cutoff point as the threshold for defining anisocoria, 30 cases were detected by the device; 12 were nonreactive. Only half (15/30) of these cases were detected by the nursing staff (Fig. 4), who also diagnosed 16 false positives.

### Quantitative versus standard assessment of PLR

**Fig. 5:**
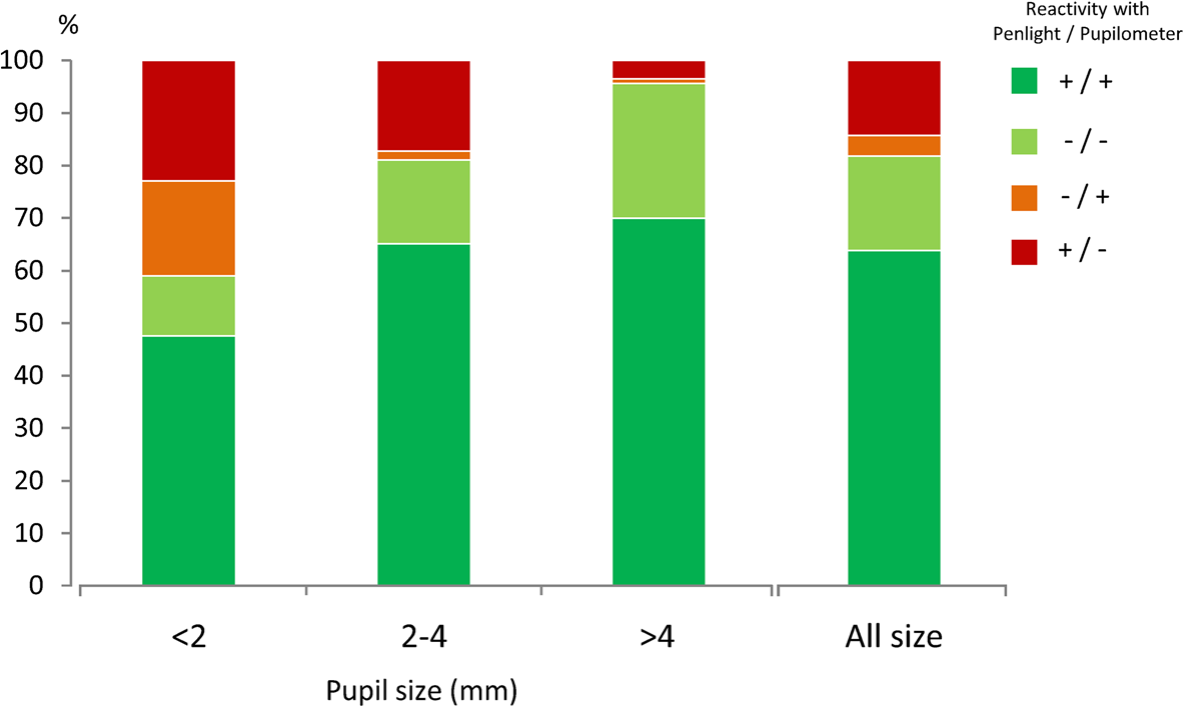
Assessment of PLR by nursing staff. Percentage agreement (*green* and *light green*) or discrepancy (*red* and *orange*) in the assessment of PLR either by standard visual examination or using the automated pupillometer. The presence (+) or absence (–) of PLR was evaluated by nurses using a penlight and by physicians using a calibrated light stimulus delivered by the pupillometer. A global rate of discordance (*red* and *orange*) of 18 % (72/406) was found between the two techniques when assessing the PLR. For measurements with small pupils (diameters <2 mm) the error rate was at its greatest: 39 % (24/61). (Color figure online)

Assessment of PLR showed that the global rate of discordance between the nurses’ estimation and the device was equal to 18 % (72/406) (Fig. 5). Discordance increased to 39 % (24/61) for small pupils (<2 mm group) and decreased to 4 % (5/122) for large pupils (over 4 mm). The rate of discordance was 19 % (43/223) for pupil sizes >2 mm and ≤4 mm, our largest test group. Among the 43 errors in this group, 41 arose where nurses’ recorded a reactivity that was not detected by the pupillometer (<15 % decrease threshold). The median amplitude of PLR measured by the pupillometer for these errors was below 0.3 mm (0.27 mm, IQR 0.22–0.38 mm) and corresponded to an 80 % decrease of the normal PLR (median PLR amplitude of 1.36 mm (IQR 1.2–1.47 mm) in healthy volunteers for pupil sizes >2 mm and ≤4 mm). We did not find any correlation between sufentanil doses and PLR (Spearman’s rho = –0.17, 95 % CI: –0.30 to 0.01; *P* = 0.07) (see Additional file 3: Figure S3B).

## Discussion

Our results reported an inaccuracy introduced by subjective testing with a poor correlation between standard visual and automated pupillometer measurements for pupil sizes between 2 and 4 mm. NCCU nurses missed 50 % of the cases of anisocoria, and wrongly detected 16 episodes of anisocoria. The global error percentage for PLR determination was 18 %. Critically, for the intermediate pupil size (2–4 mm), which represented the majority of our patients, the error rate was still high (19 %).

The lack of precision for such an important measurement (pupil size) in the monitoring of brain-injured patients is disturbing, especially given its magnitude for the pupil sizes that we most commonly assess (>2 mm and <4 mm). Although the clinical significance of pupillometry has been widely demonstrated in the literature [14], its clinical application has been limited by its subjective nature. There exists a lack of standardization for the key parameters of pupillary dilatation, speed of reactivity, and pathologic anisocoria [15]. Our study represents, to the best of our knowledge, the largest detailed application of quantitative pupillometry in an NCCU. Although Yan et al. [16] observed in 20 liver transplant patients a great accuracy between standard visual versus automated pupillometry, our results are consistent with previous studies showing a poor correlation for pupil size examination between visual and pupillometer measurements [8, 9]. Meeker at al. [9] compared the performance of an automated, portable pupillometer to visual examination performed by physicians, residents, and nurses in a small number of sedated patients in the ICU. They concluded that visual examination had a median absolute error (0.5 mm) which was double that of the pupillometer (0.23 mm).

The percentage of undiagnosed anisocoria by nurses was 50 % in our study. In their trial, Taylor et al. [14] showed that nurses only detected pupillary asymmetry in 22 % of cases. Their higher error rate can be attributed to the 0.5 mm cutoff point they used to score anisocoria. They reported this asymmetric threshold for head-injury patients with an ICP >30 mmHg, and mass effect and midline shift of >5 mm. Pupil asymmetry of less than 0.5 mm is difficult to detect by visual assessment. In addition, Boev et al. [12] showed that an asymmetry of up to 0.5 mm was frequently observed in healthy volunteers and patients without head injury. We chose 1 mm as a cutoff point for our definition of anisocoria because it is an internationally recognized threshold and is the most clinically relevant definition at the patient bedside. Chesnut et al. underscored the clinical relevance of anisocoria in their study of 210 patients with pupillary asymmetry of ≥1 mm. Sixty-three patients (30 %) had intracranial mass lesions, 52 (25 %) of which were extra-axial in location, with 38 (73 %) located ipsilateral to the larger pupil [2]. Anisocoria and asymmetrically decreased light reflexes on automated pupillometer examination can also be indicative of uncal herniation [10]. We were surprised by the high number of false-positive episodes of anisocoria diagnosed by nursing staff and the potential that this gives for unnecessary treatment or brain imaging for neurologically injured patients.

The overall 18 % error rate for the assessment of PLR is of serious concern given the fundamental nature of this measurement for establishing patient health. PLR is the most important pupil examination parameter in the routine health care of the brain-injured patient. Worse still, the majority of these misinterpretations occurred when nurses detected false-positive events that were not recorded by the pupillometer. This could be due to the cutoff point of 15 % for PLR definition (i.e., 0.6 mm in a 4 mm pupil). Our results are consistent with a previous report [14]. The mean percentage of reduction in pupil size after light stimulation was 36–40 % in adult healthy volunteers (i.e., 1.44–1.6 mm in a 4 mm pupil). Moreover, Larson and Muhiudeen [17] showed that routine clinical evaluation with traditional penlight is unable to detect the presence of a PLR when the amplitude is <0.3 mm. Taylor et al. [14] showed that the mean percentage of reduction in pupil size in brain-injured patients was 19 ± 3 % when ICP was under 20 mmHg but decreased to lower than 10 % in six patients when the ICP exceeded 30 mmHg. We choose this cutoff point (15 %) to be conservative because it represents normal visual acuity for the human eye (0.3 mm with 1 m distance and 15 % of contraction for 2 mm pupil size) [13]. The clinical implication and the significance of pupillary changes on overall outcome have been demonstrated previously [4, 18]. Morris et al. [5] reported in patients sustaining severe traumatic brain injuries that loss of pupillary activity or development of pupillary asymmetry >2 mm was correlated with higher morbidity and mortality rates. In our study, the error rate for PLR measurements was dramatically increased for small pupils (<2 mm) (39 %), which is not surprising given their small variation in absolute diameter (0.3 mm of pupil contraction with 15 % as a cutoff point). The infusion of narcotics might increase the error rate by reducing the pupil size. However, there was no difference in PLR agreement in patients with or without narcotics in our study. This is in line with the literature showing no effect of narcotics on pupil light reflex [19, 20].

Our results are in agreement with earlier reports [10, 17], and are consistent with the large pupillary size fluctuations that occur for mid-sized pupils (approximately 5 mm in diameter) versus minimal fluctuation for larger and smaller pupils [17]. The capacity of the pupillometer to detect changes in pupillary reaction even in small pupils is potentially important [21]. These devices could help to detect brain herniation in its early stages, before pupillary dilatation becomes apparent.

In critical care, automated pupillometry has been studied as a tool to measure pain during treatment compared with hetero-evaluation scales (Behavioral Pain Score) [22, 23]. But there are few studies that have examined automated pupillometry as a tool for monitoring PLR in brain-injured patients [17, 24]. It has been shown in one preliminary study [14] that PLR was impaired in brain-injured patients compared with healthy subjects; low reflex amplitude, longer latency, and a reduced speed of constriction were noted. These abnormalities could be related to intracranial hypertension. In a recent trial, Suys et al. [25] showed that automated quantitative PLR was superior to standard pupillary examination in predicting poor 90-day outcome after cardiac arrest, irrespective of hypothermic conditions and sedation, and has a comparable prognostic accuracy with electrophysiological tests, including electroencephalography and somato-sensory evoked potentials. Many parameters can be analyzed by a pupillometer in brain-injured patients, such as latency and constriction velocity. Chen et al. [26] introduced the Neurological Pupil Index (NPI) in a neurocritical care algorithm (including size, latency, constriction velocity, and dilatation velocity). They concluded that quantitative measurement and classification of pupillary reactivity using the NPI might be a useful tool in the early management of patients with increased ICP. Further research is needed to study the changes of parameters such as reactivity latency and speed contraction in the management of brain-injured patients.

This study has several limitations. First, while automated pupillometry provides a calibrated light stimulation, the penlight may give variable amounts of illumination. While this could be seen as a bias, it nonetheless reflects actual critical care practice. Secondly, we tried to limit the delay between measurements taken by the nurse and the physician to <5 minutes. This parameter may have introduced some degree of imprecision to our clinical measurements. Thirdly, we defined a 15 % threshold for PLR, which is debatable. The most accurate threshold for the definition of nonreactive pupils has not been established in the literature. This would require a large trial assessing sensitivity and specificity of PLR for a specified clinical outcome. Finally, our trial has not been designed nor powered to study the clinical impact of the automatic pupillometer use on patient’s outcome. We noted in 12 cases (see Additional file 5: Table S1) that the pupillometer changed patients’ medical management. Whether this measure may change clinical outcome has to be addressed in a larger prospective, double-blind, interventional study.

## Conclusion

Pupillary evaluations obtained subjectively at the patient’s bedside were inaccurate compared with those obtained with an automatic quantitative pupillometer device. This device can record reliable pupillary measurements. The significant error rate in detection of anisocoria by the current standard examination suggests inclusion of the automated pupil measurements in the routine health care of brain-injured patients. However, the impact of a pupillometer use on patients’ outcome has to be demonstrated in further prospective studies.

## Key messages

- Accuracy of standard practices in pupillary monitoring by nurses is poor.
- Trained nurses in NCCUs did not detect 50 % of anisocoria.
- Many undiagnosed anisocoria concerned pupil size above 4 mm and left and right pupil size difference up to 1.5 mm.
- Global error for PLR determination was about 20 %.

## Additional files

Additional file 1: Figure S1. Showing **A** the NeuroLight Algiscan quantitative pupillometer (IDMED, Marseille, France), **B** pupillary reactivity assessment using the electronic pupillometer showing the real-time automatic detection of the pupil (*green circle*) with its corresponding size (2.92 mm), **C** liquid crystal display screen after the assessment of the PLR showing the pupillogram with the three phases of pupillary reaction: **1** the pupillometer measures the pupil during a 200-millisecond period to determine the baseline pupil size (*yellow line*), **2** then starts the light stimulus during a 1-second period, and **3** measures the pupil reactivity curve over the subsequent 3 seconds to determine the minimal pupil size (*white line*). (PDF 9086 kb) Additional file 2: Figure S2. Showing pupillary asymmetry in healthy volunteers. *Circles* represent the difference between left and right pupil size (abscissa) and the mean left and right pupil size (ordinate) measured by the monocular pupillometer in 200 healthy volunteers. (PDF 89 kb) Additional file 3: Figure S3. Showing maximum resting pupil size **A** and percentage of reduction in pupil size after light stimulation **B** measured with a pupillometer as a function of the average hourly sufentanil dosing requirement. (PDF 73 kb) Additional file 4: Figure S4. Showing Bland–Altman plots for difference in pupil size estimates by nurses and automated pupillometer. *Solid line*, mean difference (bias); *dotted lines*, limit of agreement (bias ± 1.96 standard deviation). (PDF 52 kb) Additional file 5: Table S1. Presenting the 12 cases found with a specific medical management according to anisocoria detection. (PDF 388 kb)

## Abbreviations

CI: Confidence interval
CoV: Coefficient of variation
ICP: Intracranial pressure
IQR: Interquartile range
NCCU: Neurocritical care unit
NPI: Neurological Pupil Index
PLR: Pupillary light reflex
ROC: Receiver operating characteristic
SAPS2: Simplified Acute Physiology Score 2
