# Supplementary material for: Reliability of standard pupillometry practice in neurocritical care: an observational, double-blinded study
Source: Crit Care. 2016 Mar 13;20:99. doi: 10.1186/s13054-016-1239-z (PMC4828754; doi:10.1186/s13054-016-1239-z)

Figure S1: A) NeuroLight Algiscan quantitative pupillometer (IDMED, Marseille, France); B) Pupillary reactivity assessment using the electronic pupillometer showing the real-time automatic detection of the pupil (green circle) with its corresponding size (2.92 mm); C) Liquid crystal display screen after the assessment of the PLR showing the pupillogram with the three phases of pupillary reaction: 1) the pupillometer measures the pupil during a 200-milliseconds period to determine the baseline pupil size (yellow line); 2) then starts the light stimulus during a 1-second period and 3) measures the pupil reactivity curve over the subsequent 3-seconds to determine the minimal pupil size (white line).

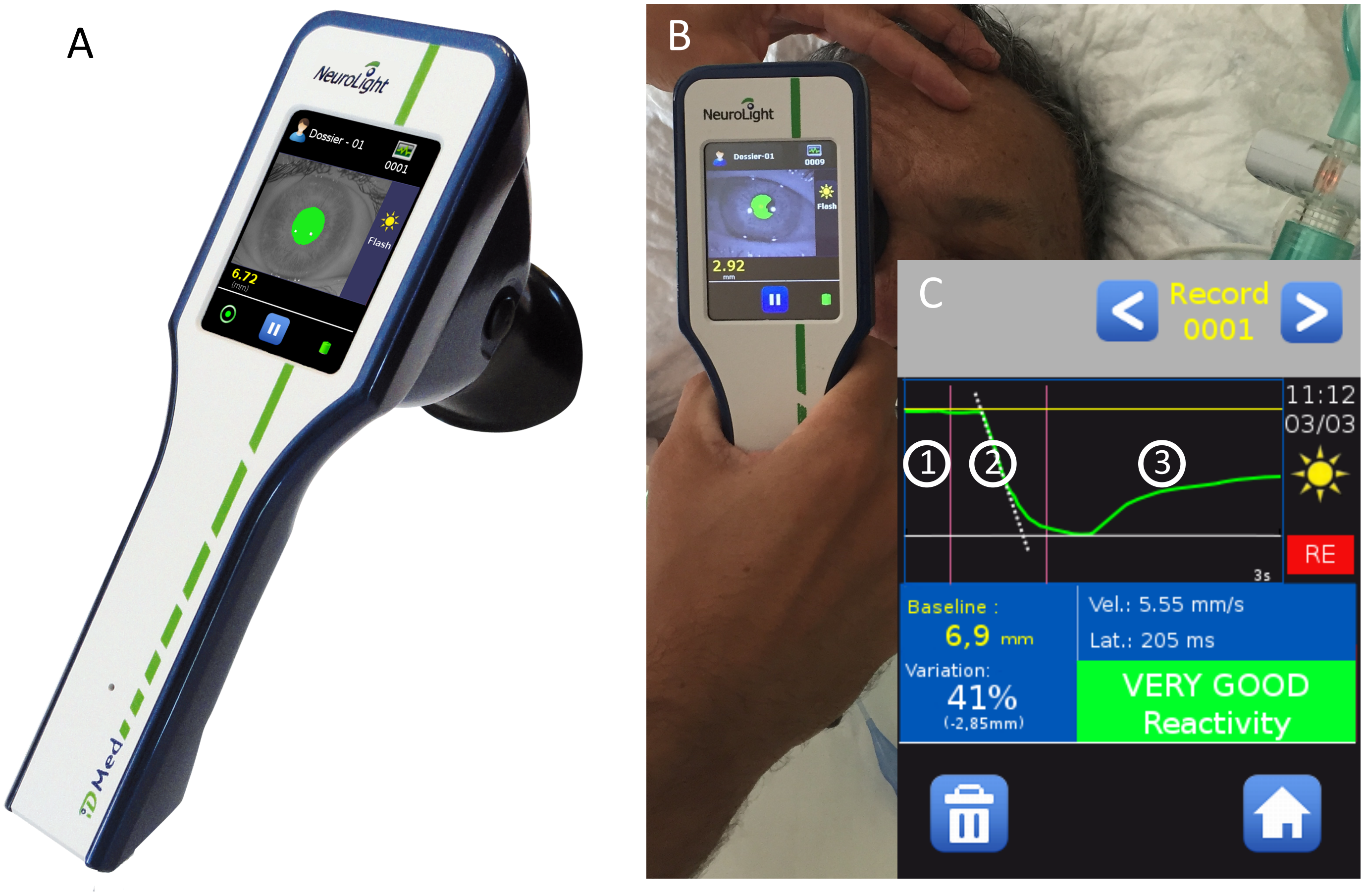

Supplement: Additional file 1: Figure S1. — Showing A the NeuroLight Algiscan quantitative pupillometer (IDMED, Marseille, France), B pupillary reactivity assessment using the electronic pupillometer showing the real-time automatic detection of the pupil (green circle) with its corresponding size (2.92 mm), C liquid crystal display screen after the assessment of the PLR showing the pupillogram with the three phases of pupillary reaction: 1 the pupillometer measures the pupil during a 200-millisecond period to determine the baseline pupil size (yellow line), 2 then starts the light stimulus during a 1-second period, and 3 measures the pupil reactivity curve over the subsequent 3 seconds to determine the minimal pupil size (white line). (PDF 9086 kb) [file 13054_2016_1239_MOESM1_ESM.pdf]
