# Supplementary material for: Reliability of standard pupillometry practice in neurocritical care: an observational, double-blinded study
Source: Crit Care. 2016 Mar 13;20:99. doi: 10.1186/s13054-016-1239-z (PMC4828754; doi:10.1186/s13054-016-1239-z)

Figure S2: Pupillary asymmetry in healthy volunteers. Circles represent the difference between left and right pupil size (abscissa) and the mean left and right pupil size (ordinate) measured by the monocular pupillometer in 200 healthy volunteers.

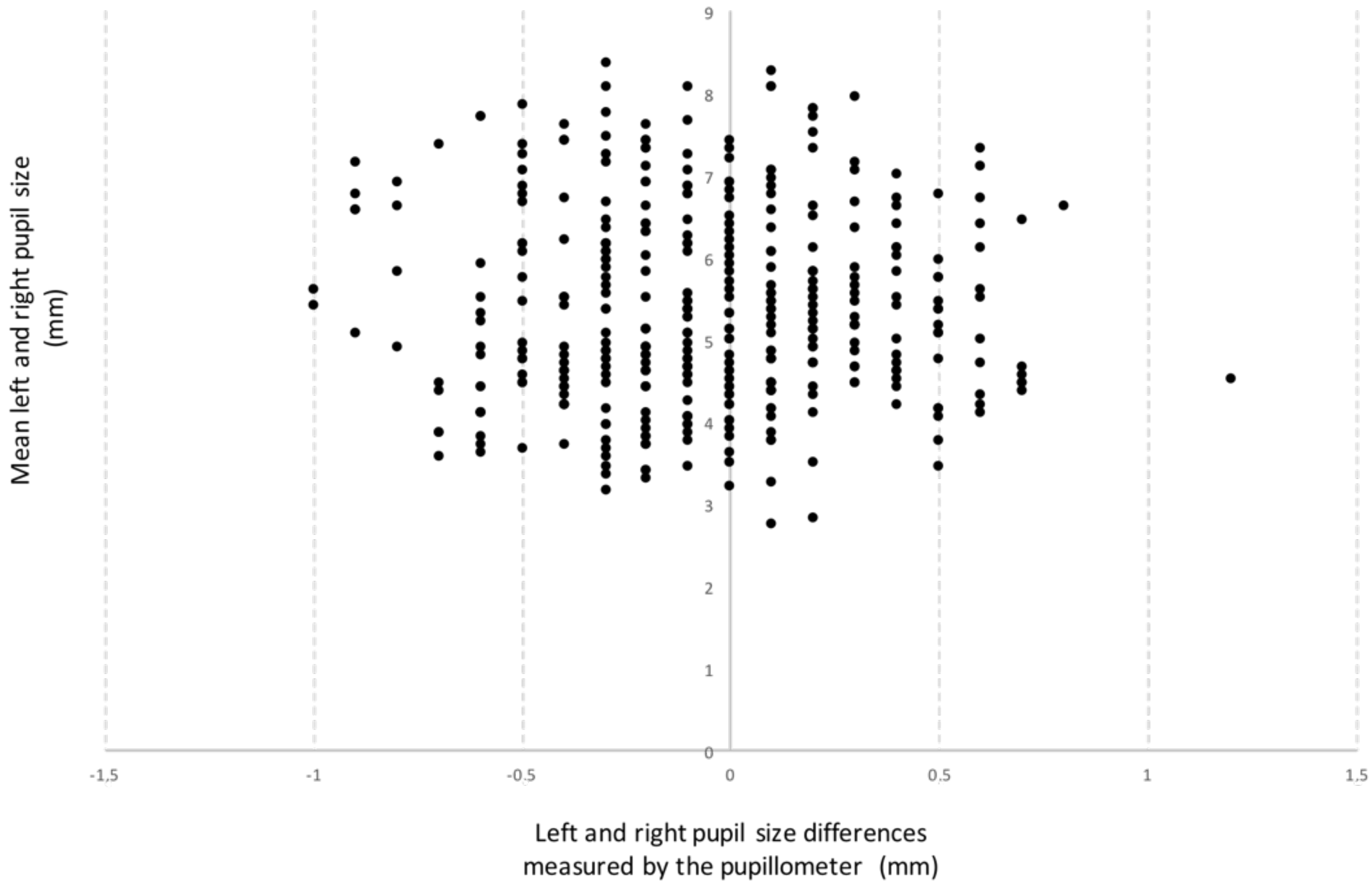

Supplement: Additional file 2: Figure S2. — Showing pupillary asymmetry in healthy volunteers. Circles represent the difference between left and right pupil size (abscissa) and the mean left and right pupil size (ordinate) measured by the monocular pupillometer in 200 healthy volunteers. (PDF 89 kb) [file 13054_2016_1239_MOESM2_ESM.pdf]
