# Supplementary material for: Reliability of standard pupillometry practice in neurocritical care: an observational, double-blinded study
Source: Crit Care. 2016 Mar 13;20:99. doi: 10.1186/s13054-016-1239-z (PMC4828754; doi:10.1186/s13054-016-1239-z)

Figure S3: Maximum resting pupil size (A) and percentage of reduction in pupil size after light stimulation (B) measured with pupillometer in function of the average hourly sufentanil dosing requirement.

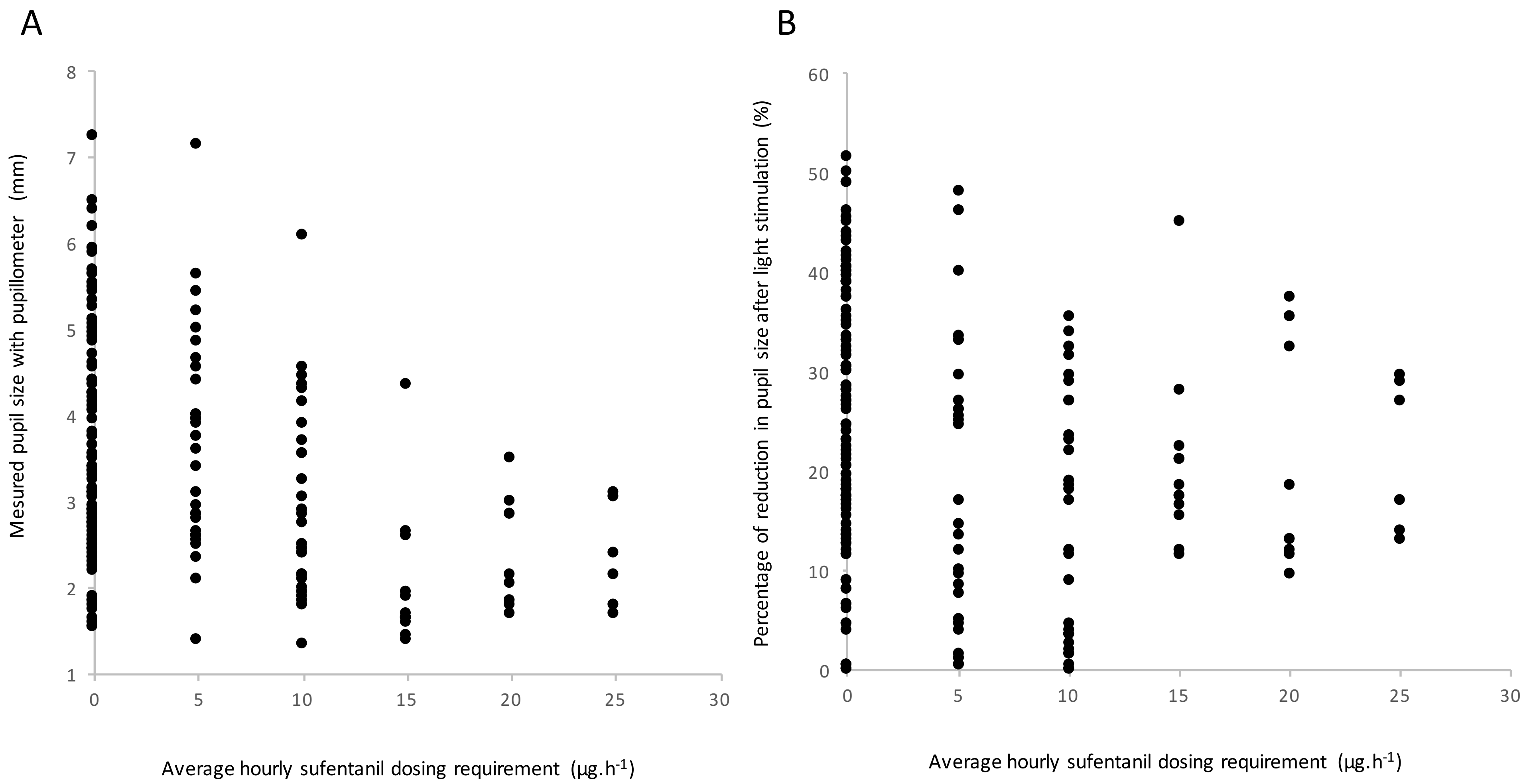

Supplement: Additional file 3: Figure S3. — Showing maximum resting pupil size A and percentage of reduction in pupil size after light stimulation B measured with a pupillometer as a function of the average hourly sufentanil dosing requirement. (PDF 73 kb) [file 13054_2016_1239_MOESM3_ESM.pdf]
