# Supplementary material for: Reliability of standard pupillometry practice in neurocritical care: an observational, double-blinded study
Source: Crit Care. 2016 Mar 13;20:99. doi: 10.1186/s13054-016-1239-z (PMC4828754; doi:10.1186/s13054-016-1239-z)

Figure S4: Bland–Altman plots for difference in pupil size estimates by nurses and automated pupillometer. Solid line, mean difference (bias); dotted lines, limit of agreement (bias± 1.96 SD).

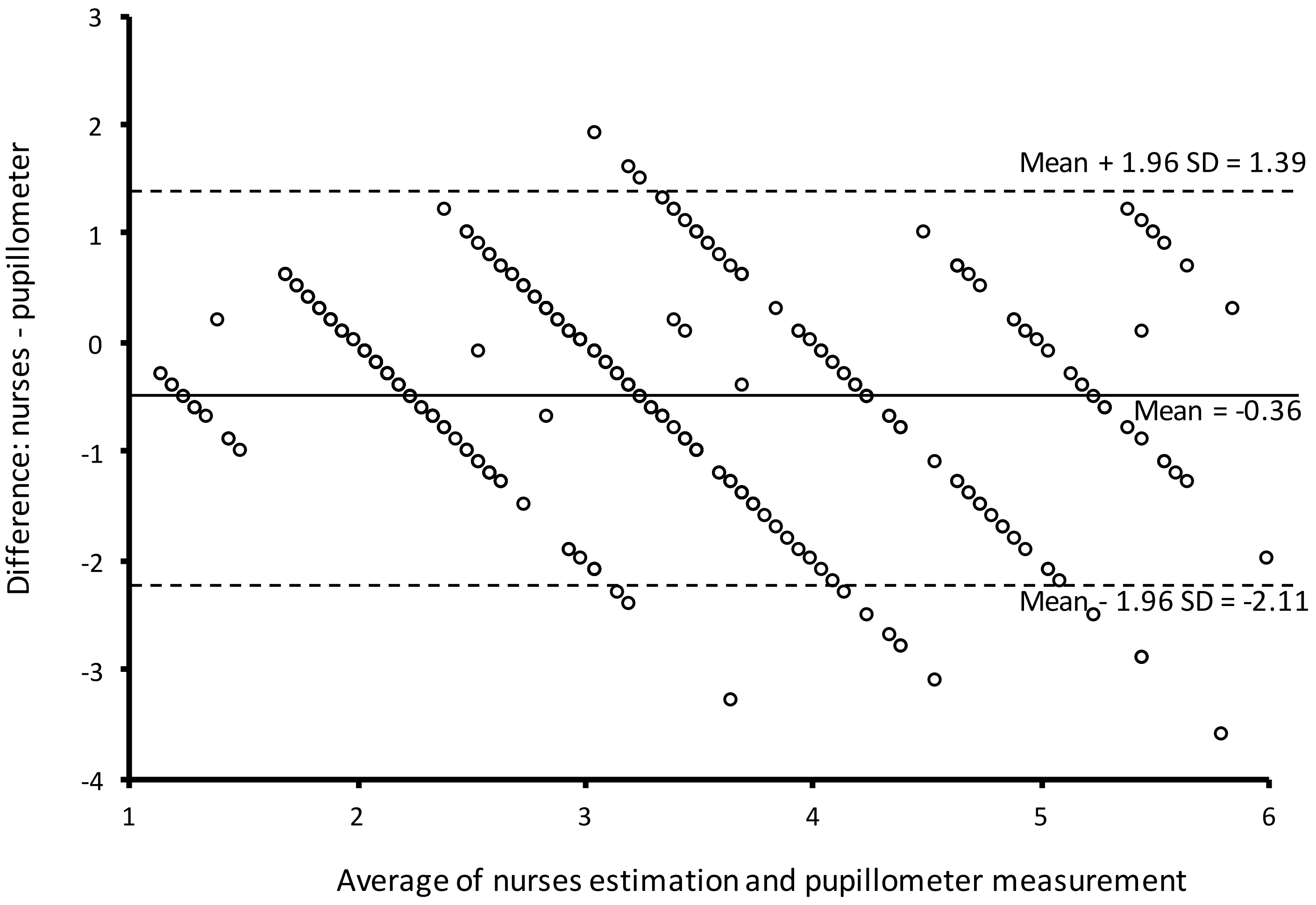

Supplement: Additional file 4: Figure S4. — Showing Bland–Altman plots for difference in pupil size estimates by nurses and automated pupillometer. Solid line, mean difference (bias); dotted lines, limit of agreement (bias ± 1.96 standard deviation). (PDF 52 kb) [file 13054_2016_1239_MOESM4_ESM.pdf]
