# Supplementary material for: Reliability of standard pupillometry practice in neurocritical care: an observational, double-blinded study
Source: Crit Care. 2016 Mar 13;20:99. doi: 10.1186/s13054-016-1239-z (PMC4828754; doi:10.1186/s13054-016-1239-z)
Supplement: Additional file 5: Table S1. — Presenting the 12 cases found with a specific medical management according to anisocoria detection. (PDF 388 kb) [file 13054_2016_1239_MOESM5_ESM.pdf]

Table S1. We found 12 cases with a specific medical management according to anisocoria detection.

Cases	Age	Sex	Causes of NCCU admission	GCS admission	Opioid infusion when anisocoria daignosed	Sedative infusion when anisocoria	Clinical symptoms	Pupil asymmetry (mm)	Disagreement for anisocoria	Type of anisocoria	Intervention	Imaging results	ICP (mmHg)	mRS outcome
**1**	20	M	Traumatic brain injury	12	-	-	Confusing state	1.1	Anisocoria detected by pupillometer	Reactive	Sedation with propofol and urgent CT brain scan	Increased edema and midline shift	-	0
**2**	61	M	Decompressive craniectomy for malignant infarction of the right middle cerebral artery	3	-	-	Altered consciousness	1.3	Anisocoria detected by pupillometer	Reactive	Sedation with propofol and urgent CT brain scan	Increased cerbral hernia and midline shift	-	3
**4**	61	F	Spontaneous cerebral haematoma	7	+	+	Non evaluable due to therapeutical sedation	0.2	Anisocoria detected by nurse	Areactive	Intracranial pressure monitorage		5	6
**5**	40	F	Subarachnoid hemorrhage	15	-	-	Delayed ischemic neurological deficit	1.8	Anisocoria detected by pupillometer	Areactive	Urgent MRI	Left temoral Ischemia	-	1
**6**	18	F	Mesencephalic pilocytic astrocytoma	10	+	+	Altered consciousness with aspiration pneumonia	1.1	Anisocoria detected by pupillometer	Areactive	Neursurgeon examination	NA	-	4
**7**	15	F	Traumatic Brain Injury	5	+	+	Non evaluable due to therapeutical sedation	0.4	Anisocoria detected by nurse	Areactive	CT scan	Right temporal contusion	8	5
**8**	73	M	Intra cranial hemmorhage under AVK	9	-	-	Altered consciousness	0.6	Anisocoria detected by nurse	Reactive	Neurosurgeon examination	0	-	5
**9**	56	M	Subarachnoid hemorrhage	15	-	-	Altered consciousness	0.2	Anisocoria detected by nurse	Areactive	Ct scan declampage	Hydrocephaly	-	6
**10**	61	M	Frontal Abcess	3	-	+	No evaluable due to therapeutical sedation	0.3	Anisocoria detected by nurse	Areactive	Sedative infusion and CT scan Mannitol before surgical cure	Frontal abcess with subfalcine herniation	-	1
**11**	57	M	Intra cranial Hemorrhage	3	-	-	Altered consciousness	0.5	Anisocoria detected by nurse	Areactive	Sedative infusion, CT scan	Rebleeding with mass effect	-	6
**12**	64	F	Spontaneous cerebral haematoma	3	+	+	Non evaluable due to therapeutical sedation	1.2	Anisocoria detected by pupillometer	Areactive	Urgent CT scan	Hemorrhage growth with mesencephalon infarction	25	6
